# Repeating Spatial-Temporal Motifs of CA3 Activity Dependent on Engineered Inputs from Dentate Gyrus Neurons in Live Hippocampal Networks

**DOI:** 10.3389/fncir.2016.00045

**Published:** 2016-06-28

**Authors:** Aparajita Bhattacharya, Harsh Desai, Thomas B. DeMarse, Bruce C. Wheeler, Gregory J. Brewer

**Affiliations:** ^1^Department of Biomedical Engineering, University of CaliforniaIrvine, CA, USA; ^2^J. Clayton Pruitt Family Department of Biomedical Engineering, University of FloridaGainesville, FL, USA; ^3^Department of Pediatric Neurology, University of FloridaGainesville, FL, USA; ^4^Department of Bioengineering, University of CaliforniaSan Diego, CA, USA; ^5^Memory Impairments and Neurological Disorders (MIND) Institute, University of CaliforniaIrvine, CA, USA

**Keywords:** networks, electrode array, dentate, CA3, microtunnels, motifs, burst

## Abstract

Anatomical and behavioral studies, and *in vivo* and slice electrophysiology of the hippocampus suggest specific functions of the dentate gyrus (DG) and the CA3 subregions, but the underlying activity dynamics and repeatability of information processing remains poorly understood. To approach this problem, we engineered separate living networks of the DG and CA3 neurons that develop connections through 51 tunnels for axonal communication. Growing these networks on top of an electrode array enabled us to determine whether the subregion dynamics were separable and repeatable. We found spontaneous development of polarized propagation of 80% of the activity in the native direction from DG to CA3 and different spike and burst dynamics for these subregions. Spatial-temporal differences emerged when the relationships of target CA3 activity were categorized with to the number and timing of inputs from the apposing network. Compared to times of CA3 activity when there was no recorded tunnel input, DG input led to CA3 activity bursts that were 7× more frequent, increased in amplitude and extended in temporal envelope. Logistic regression indicated that a high number of tunnel inputs predict CA3 activity with 90% sensitivity and 70% specificity. Compared to no tunnel input, patterns of >80% tunnel inputs from DG specified different patterns of first-to-fire neurons in the CA3 target well. Clustering dendrograms revealed repeating motifs of three or more patterns at up to 17 sites in CA3 that were importantly associated with specific spatial-temporal patterns of tunnel activity. The number of these motifs recorded in 3 min was significantly higher than shuffled spike activity and not seen above chance in control networks in which CA3 was apposed to CA3 or DG to DG. Together, these results demonstrate spontaneous input-dependent repeatable coding of distributed activity in CA3 networks driven by engineered inputs from DG networks. These functional configurations at measured times of activation (motifs) emerge from anatomically accurate feed-forward connections from DG through tunnels to CA3.

## Introduction

Since the hippocampus is critically important to cognitive memory formation, a long history of research has elucidated detailed information ranging from the molecular and cellular to the systems and behavioral levels. However, much remains to be learned in between the macro and single cell levels, more specifically as to how subregions of the hippocampus encode the information thought to propagate along the trisynaptic circuit from entorhinal cortex (EC) to dentate gyrus (DG) to areas CA3 and CA1. While microelectrodes and arrays have been used to monitor activity in behaving animals and in brain slices, with attendant advantages and problems, there remains a need for models more uniquely enabling the manipulation and monitoring of the connectivity between the subregions.

**Figure 1:**
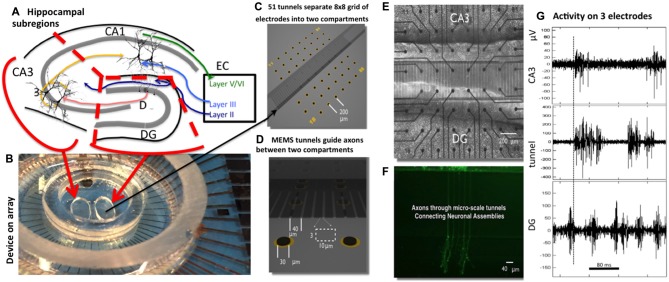
**Two-chamber Micro-Electro-Mechanical Systems (MEMS) device for reconstruction of hippocampal subregional circuits on a multi-electrode array. (A)** Subregions in a slice of rat hippocampus (dissection follows red lines). **(B)** 60 electrode array with two chamber MEMS device. **(C)** Distribution of 22 electrodes in each chamber separated by 51 tunnels of 400 μm length aligned to the eight columns of electrodes. **(D)** Tunnel 3 × 10 μm cross-section aligned over an electrode pair (black oval). **(E)** Phase contrast imaging of live neurons over electrode array at 3 weeks. **(F)** Fluorescent labeling shows selective growth of axons from one compartment into another. **(G)** Sample recordings of high-pass filtered signals from one electrode in dentate gyrus (DG), tunnel and CA3 compartment. The dashed line marks the start of activity on the tunnel electrode, about 5 ms before activity on the CA3 electrode, both after substantial activity on the DG electrode. Note larger amplitudes for spikes in the tunnel.

To that end, we have developed a microfabrication (Micro-Electro-Mechanical Systems or MEMS) technology in which subregions of live hippocampal neurons are cultured in separate compartments that appropriately connect by axons traversing microtunnels (Figure 1) after Taylor et al. (2003), Claverol-Tinturé et al. (2007) and Wang et al. (2012). We have previously shown that this technology supports hippocampal subregion-specific growth in separate compartments of tissue from pairs of the DG, CA3 and CA1 regions, with the emergence of appropriate functional feed-forward axonal connections (Brewer et al., 2013). The model was validated by showing that *in vivo* molecular markers are appropriately and differentially expressed in the three tissue subregions after dissection, isolation, and regrowth in culture. Further, the model was shown to exhibit elementary “self-wiring”, namely an appropriate directional communication preference with more axons propagating spikes from DG to CA3 than in reverse, consistent with understanding of propagation of information from DG to CA3 *in vivo*. The system also exhibits modulation of firing and burst rates as a function of the number of axon tunnel connections of similarly cultured cortical networks (Pan et al., 2015). Our results showed that as *in vivo*, the *in vitro* DG-CA3 circuit is preferentially feed-forward, with more inhibitory neuron involvement in DG and astroglial control in CA3, thus fitting classic models of neural and information processing.

Thus, these results provided encouragement for further exploration of the suitability of this experimental system as a model for brain and in particular hippocampal functioning. We do this by determining whether the spatial and temporal dynamics of spontaneous activity is modulated at each step of hippocampal processing, i.e., separable and repeatable. More specifically we report here the investigation of how, in this model, the anatomical and functional target of inputs in the CA3 subregion is impacted by its appropriate input from DG. Straightforwardly, we expect that DG input should participate in eliciting responses in CA3. At a more detailed level are the many follow-on questions: is bursting in CA3 spatially or temporally different when stimulated by DG than when spontaneously generated? Are there differences in burst intensity and duration when driven by DG and do all bursts begin at the same locations (as modulated by “leader neurons”)? The answers are helpful in understanding the validity of our *in vitro* hippocampal model as well as for expanding our understanding of how these essential brain subregions encode information.

As *in vivo*, hippocampal and cortical neurons *in vitro* develop into networks with prominent bursting activity (Maeda et al., 1995, 1998; van Pelt et al., 2004; Wagenaar et al., 2006). In homogenous networks, leader neurons or ignition sites can be identified whose spontaneous activity often initiates a burst of spikes that propagate through the network comprising a network burst (Eytan and Marom, 2006; Ham et al., 2008). Temporal sequences in rat cortical cultured networks were first demonstrated by cross correlation and sorted into spatial groups with specifically ordered firing or motifs (Segev et al., 2004; Volman et al., 2005). A motif is a structural assembly of neurons in a network, a subset of which is functional at particular times (Sporns and Kötter, 2004; Poli et al., 2015). Thus, a repeating motif could be observed as a repeated temporal pattern of activity in one or more neurons, or a spatial pattern of activity repeated in time. Early examples include Ikegaya et al. (2004) synfire chains in mouse slices and Rolston et al. (2007) dissociated rat cortical culture. These motifs can also be detected by synching to the time and location of the first burst after a quiescent period (Stegenga et al., 2008) to make temporal predictions of the spreading pattern of activity in cortical networks (Eckmann et al., 2008). Here we examine the origins of the bursting activity in a 2-compartment device containing a network of DG neurons separated by 400 μm tunnel pathways before reaching CA3 neurons (Figure 1). Our tunnel device allows access to the inputs that elicit the bursts in the target well. The tunnels are similar to those of Taylor and Jeon (2011), but the device was placed over a 60 electrode array to be able to monitor the neuronal activity. This type of system was used by Bisio et al. (2014) to follow the development of bursting activity over several weeks and transmission from one compartment to another. A two-compartment device for separate brain regions was first reported by Kanagasabapathi et al. (2013) to show bi-directional communication between thalamic and cortical networks, with bursts primarily initiated in the cortical side. Here we not only report spontaneous repeated spatial patterns but show how they are dependent on the quantity and timing of the inputs from the network in the opposing chamber.

## Materials and Methods

### Neuronal Network Culture

Harvesting and dissection of individual hippocampal subregions is described in more detail here (Brewer et al., 2013). Briefly, hippocampal sub-regions were isolated from postnatal day 3 rats to obtain the neurons. The entire hippocampus was removed intact from the overlying neocortex of each hemisphere before further sub-region dissection. First CA1 was separated from DG-CA3 by making cuts along the DG-CA1 boundary using DG rostral and ventral ends as anchors. Then CA3 was separated from DG using clearly visible boundaries under the microscope.

Cells from each hippocampal sub-region were plated on poly-D-lysine coated MEAs with attached PDMS micro-tunnels in NbActiv4^TM^ medium (Brewer et al., 2008; BrainBits, Springfield, IL, USA). Poly-D-lysine (Sigma SLBB8061V) was dissolved at 37°C for 1 h in sterile water before application to the devices at 100 μg/mL and incubation overnight at room temperature. Well dimensions were ovoid with 1 mm height and volume of 6.3 mm^3^. DG cells were plated at a density of 1000 cells/mm^2^ in one well (the bottom) and CA3 cells were plated in the other well (the top) at 330 cells/mm^2^ to mimic the *in vivo* anatomical density ratio of 3:1 for DG-CA3 (Braitenberg, 1981). PDMS tunnels promoted growth of axons between the sub-region cells. Each of the 51 tunnels were 400 μm long, 10 μm wide, 3 μm height, and 40 μm apart such that they covered seven electrode pairs in the middle of the MEA. Each well was positioned over 22 of the 59 electrodes in an 8 × 8 grid shown in Figures 1C,E (Multi Channel Systems, Reutlingen, Germany). For demonstration of axon transmission through the tunnels, DG neurons in one compartment were labeled for 20 min with 2 μM Calcein-AM (Molecular Probes, Carlsbad, CA, USA) and imaged with a confocal microscope. Control homologous cultures of DG-DG were plated at 1000 cells/mm^2^ and CA3-CA3 were plated at 330 cells/mm^2^. DG cells were plated first in the bottom well, and incubated for 15–30 min before plating the target cells in the top well. Our group reported that a 7 day delay in seeding a second well with CORTICAL neurons achieved significant polarization of directionality from the first to the second compartment (Pan et al., 2011). In our work with hippocampal neurons (Brewer et al., 2013), as here, intrinsic neuronal properties promote the native directionality of DG to CA3 even with nearly simultaneous plating. Incubation conditions were 37°C, 5% CO_2_, 9% O_2_ and saturating humidity (Thermo-Forma #3432, Columbus, OH, USA) with evaporation limited by covering the network with a Teflon film (ALA Scientific, *Farmingdale*, NY, USA). One-half of the culture medium was removed and replaced with the same volume of fresh medium every 4–5 days.

### Multi-Electrode Array and Recording

Activity from each multielectrode array MEA60 (Multichannel Systems, Reutlingen, Germany; ALA Scientific, Farmingdale, NY, USA) was recorded at 25 kHz sampling frequency with 1100× amplification and a hardware filter of 1-3000 Hz. The recordings were made at 37°C under continuous flow of hydrated sterile 5% CO_2_, 9% O_2_ and balance N_2_ (Airgas custom, Santa Ana, CA, USA) over a Teflon film cover (ALA Scientific). Three minutes of spontaneous activity was recorded using Multichannel System’s MCRack software and analyzed offline using custom Matlab scripts.

### Spike Activity Analysis

#### Spikes/Burst Detection and Dynamics

The digitized signal from each electrode was first filtered using a 300-Hz high-pass filter followed by spike detection. Spikes were identified as peak-to-peak voltages exceeding 11× the minimum root-mean-square value of 200 ms contiguous windows (Spycode v3.9; Maccione et al., 2009; Bologna et al., 2010). We imposed a 1 ms dead-time or refractory period after a spike before another could be detected. As before Brewer et al. (2013), bursts were defined as four or more spikes with an interspike interval (ISI) of 1–50 ms, on a per electrode basis, not summed over multiple electrodes. Distributions of spike and burst dynamics for each array and their combinations were accumulated using either codeResource (courtesy of Stathis Leondopulos, Princeton, NJ, USA) for log-normal distributions or plfit for log-log (power law) distributions from Clauset et al. (2009)^1^. Fitting statistics were determined for each array as the best linear fit on a log-log scale (largest L, Kolmogorov-Smirnov statistic using Clauset’s program). Statistics include alpha as the best linear slope and x min, the start of the fit. To determine whether two regions were characterized by different model slope fits of the linear region of the distribution, each paired DG and CA3 from *n* = 5 data sets were then compared by compartment using a two-tailed, paired *t*-test of slopes (alpha) with criterion set to *p* < 0.05.

#### Direction of Spike Propagation from Delays Between Two Tunnel Electrodes

Propagation delays between sequential spikes on the two neighboring microelectrodes in each tunnel were used to determine the directionality of propagation. A histogram of delays was calculated between limits of ±280–600 μs to identify the peak. The limits for delay are based on the known range of axonal conduction delays (Patolsky et al., 2006). We have termed “forward propagation” as the direction in which the spikes propagated from DG to CA3 in DG-CA3 networks and the direction of propagation for more than half of the DG-DG and CA3-CA3 networks.

#### Spike Train Processing to Determine Events and Windows of Activity

For each electrode, the spike trains were smoothed with a 32 ms Gaussian kernel, and down-sampled to 1.25 kHz. These smoothed trains were then summed across all electrodes in a well or in the tunnels for a measure of mutual activity. Thus, sporadic spikes make small and bursts make large contributions to these events. We use activity or event interchangeably to be an excursion from zero (region of no spikes) of the smoothed, regionally summed signal, with the event’s time window beginning with the first positive deviation from zero and ending when the signal returned to zero (see Supplementary Figure 1). Events were then constructed from each temporal window and spiking within each event binned at 5 ms for each electrode for postevent histograms in CA3. The entire event envelope for each electrode constituted the feature space for later motif classification by region described below.

#### Time Relationships of DG, Tunnel and CA3 Events

Events in CA3 were detected and categorized based on tunnel event timing as follows:

1. CA3 only (isolated with no temporal overlap of the activity window, within 32 ms, of the windows of events in tunnels or DG);
2. Tunnel event drives CA3, presumably from DG (the tunnel activity window begins shortly before and continues through the start of the CA3 activity window;
3. Tunnel driven by CA3, (CA3 activity begins before the start of a tunnel activity window);
4. Events which started simultaneously in both CA3 and the tunnels were fewer than 5% of total CA3 events and were ignored.

To use the DG-DG and CA3-CA3 networks as controls, we first determined in each cultured network which well had more than half of all spikes and designated this well as “target” in order to maximize target events and counter-bias against a strong driver. We then used the statistics of the events in that “target” well for comparison to the CA3 as the target in the DG-CA3 networks.

#### Leader Neurons

To determine if the target network initiated activity at certain repeatable locations, we identified the leader neuron, or, more properly, the leader electrode, as the electrode reporting the first spike in a regional activity window. Leader neuron probability was computed on a per electrode basis by dividing the number of times an electrode reported the first spike in an event by the total number of events. These probabilities were computed for the different categories of events.

#### Logistic Regression

Tunnel count, maximum spike rate, tunnel activation order, and presence or absence of tunnel activity were evaluated as potential discriminatory classifiers to predict propagation of activity from tunnels to target. A logistic regression model was fit to the response of tunnel activity propagation to target (success or failure) using one of the above classifiers as the predictor (Matlab function glmfit). Probabilities of the responses predicted from the fit (Matlab glmval) were used to plot the Receiver Operating Characteristic (ROC) curve (Matlab perfcurve).

#### Identifying Spatial Patterns of Target Activity

Dendrograms were used to classify target events into clusters with matching active electrodes. Since lower tunnel activity produced fewer target events, target events driven by six or more active tunnels (90% of activity) were chosen for this analysis. For N number of target events and 22 target electrodes, an N × 22 activity matrix of zeroes and ones was created where one denoted activity at an electrode and zero otherwise. The hierarchical cluster tree (Matlab linkage) was obtained based on Ward linkage using Euclidean distances between rows of the activity matrix (plot dendrogram function). Thus, a distance of zero indicates identity with distance increasing with increasing differences.

#### Target Activity Patterns Based on Tunnel Activity Pattern

We determined whether the pattern of tunnel activity might be predictive of which pattern of activity (i.e., motif) that emerged subsequent to tunnel activiation. To accomplish this, we first constructed an M × 8 matrix of tunnel events (M is the number of events in 8 tunnel columns) consisting of “0” for no activity, “1” for first-to-fire and “2” for second-to-fire. First-to-fire and second-to-fire electrodes were separated by 32 ms or more. Tunnel events were classified into clusters with similar activation order (Matlab linkage). Leader neuron probability in the target was computed for each tunnel cluster to determine if tunnel activation patterns were predictive of leader neuron identity. Also activity in the target was calculated by dividing the number of times an electrode was active in the target network by the total number of target events corresponding to the tunnel cluster.

## Results

The goal of this study was to understand the dynamics of spontaneous communication between two subregions of the hippocampus by reconstructing the parts in an engineered MEMS device over an electrode array (Figure 1). In this device, DG neurons isolated from postnatal day 3 rats spontaneously extend axons through the microtunnels and connect to target CA3 neurons (Brewer et al., 2013). Spontaneous neuron activity was sampled by 22 electrodes in each of the DG “source” and CA3 “target” well as well as in 8 of the 51 tunnels that connect the two-compartments after 3 weeks of development. Two-compartment devices with DG neurons in both wells, or CA3 neurons in both wells were used as homologous controls.

### Directionality of Signal Propagation

Because we have two electrodes in seven of the tunnels, we can compute delay times in spike propagation between these electrodes through the tunnels. Here we analyzed all spike pairs within 280–600 μs in paired electrodes within each tunnel. For this 200 μm distance, this restricts axon spike conduction velocities for 0.3–0.7 m/s. Granule cells in DG predominantly synapse uni-directionally with the pyramidal cells in CA3 *in vivo*. This axonal polarity of DG to CA3 is maintained in the engineered DG-CA3 networks examined in this study. 84 ±7% (*p* < 0.01) of the spike-pairs used for delay calculations showed forward conduction from DG to CA3 with a mean conduction velocity of 0.51 ± 0.13 m/s (SD, *n* = 36015 spike-pairs), confirming our earlier measure of 0.54 m/s for only isolated spikes outside of bursts (Brewer et al., 2013), despite nearly simultaneous plating times and demonstrating that a 1 week plating delay for cortical neurons (Pan et al., 2011) was not essential to achieve directionality of these hippocampal neurons. In contrast, the control networks DG-DG and CA3-CA3 with one compartment selected as “target” showed nearly equal distribution of delays indicating no preferred direction of spike propagation. Mean forward conduction velocity in DG-DG was 0.48 ± 0.13 m/s (*n* = 8642 spike-pairs) and in CA3-CA3 networks was 0.48 ± 0.11 m/s (*n* = 3291 spike-pairs), suggesting similar axon diameters in the same 10 × 3 μm cross-section tunnels and less efficiency of connection compared to the native DG-CA3.

### Spike and Burst Dynamics

**Figure 2:**
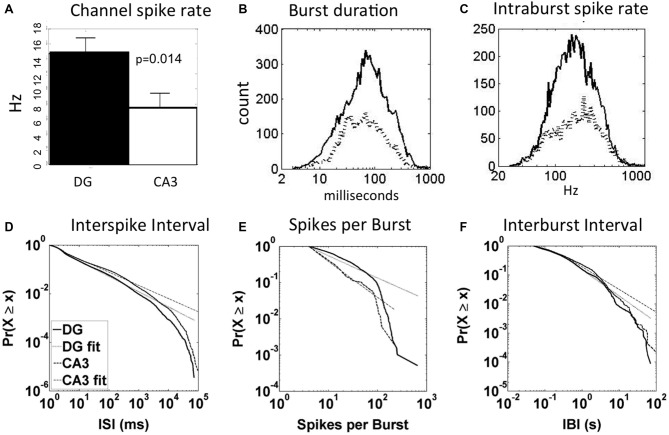
**Spike and burst dynamics for DG-CA3 networks distribute as log-normal and log-log relationships that highlight regional differences. (A)** Larger average channel spike rate in DG than CA3 (*N* = 72 electrodes in DG and 59 electrodes in CA3 from five arrays). **(B)** Log-normal higher mean burst duration (mean on x-axis) in DG (67 ms; solid line, *n* = 6817 bursts) than CA3 (58 ms; dashed line, *n* = 3386). **(C)** Lower mean intraburst spike rate in DG (solid line) than CA3 (dashed line). **(D)** Cumulative log-log fit of interspike intervals (ISI) in DG and CA3 are similar in slope over three orders of magnitude (X is normalized total counts (total count in all bins/total count = 1); small × is counts in all bins less than the current bin) *n* = 5 arrays merged with 173030 spikes in DG and 70324 in CA3. **(E)** Log-log spikes per burst are fit well over one order of magnitude with higher spike count in DG than CA3. This panel is for one representative array. Because of large differences in the log-log distributions of spikes per burst for each array, the combination was considerably rounded and not well fit by a line. **(F)** Similar slopes for interburst intervals over two orders of magnitude in DG (*n* = 173030 spikes) and CA3 (*n* = 70324). Dotted lines indicate linear regions of log-log distributions.

In our previous report on these same DG-CA3 networks (Brewer et al., 2013), we found 64% of DG subnetwork spikes occurring in bursts and only 45% of CA3 subnetwork spikes in bursts (*p* < 0.01). For burst duration and intraburst spike rates in DG and CA3, we had assumed Gaussian distributions, ignoring the long tails of events. Figure 2 here shows five aspects of spike and burst dynamics that are non-Gaussian with long tails, either symmetric log-normal or linear log-log distributions (Clauset et al., 2009; Buzsáki and Mizuseki, 2014). Figure 2A shows a higher average channel spike rate in DG (11.5 Hz) compared to CA3 (6.6 Hz), excluding channels with rates lower than 0.01 Hz, but the distribution was not Gaussian, log-normal or log-log probably because of the small n (not shown). Figure 2B shows 16% longer burst durations in DG (67 ms) than CA3 (58 ms) with symmetric log-normal distributions (*p* = 0.026). Similarly distributed intraburst spike rates (Figure 2C) were 10% lower in DG (163 Hz) than CA3 (181 Hz; *p* = 0.014). Three other measures of network dynamics exhibited somewhat linear log-log distributions. We present cumulative distributions, which have a benefit of smoothing the inevitable low sampling at the extremes of measurement (Newman, 2005). Figure 2D shows that ISI are well fit by a log-log distribution over three orders of magnitude with a 14% higher slope (*α*) in DG (*α* = 1.73 ± 0.02) than CA3 (*α* = 1.52 ± 0.01; *p* = 0.04 by paired *t*-test, all intervals from *n* = 5 arrays). Although spikes per burst were well fit by a log-log distribution in each array (Figure 2E), more rounded distributions resulted from combining arrays with different ranges and an insignificant difference in slope (DG *α* = 2.68 ± 0.15, CA3 *α* = 2.30 ± 0.07). Similar rounding was seen in the combined log-log distributions of interburst intervals (Figure 2F), which were generally linear from 0.05 to 5 s and did not differ between regions (DG *α* = 1.90 ± 0.47, CA3 *α* = 1.78 ± 0.40). Together these basic spike and burst dynamics indicate lognormal distributions for burst duration and intraburst spike rates. Bursts were longer with slightly lower spike rates in DG than CA3. The log-log distributions of ISI are not meaningfully characterized with a mean, but the higher alpha in DG than CA3 indicates fewer short ISI, consistent with lower intraburst spike rates (Figure 2C). Other measures of alpha for interburst spike rate and spikes per burst were not significantly different. Note that within any band of spike or burst frequencies, an infinite range of precisely timed spike patterns could transmit different information, even when the average statistics are the same (e.g., Sandler et al., 2015). In the following, we identify some of the specifying input and output characteristics.

### Effect of Tunnel Inputs on CA3 Activity Amplitude and Timing

**Figure 3:**
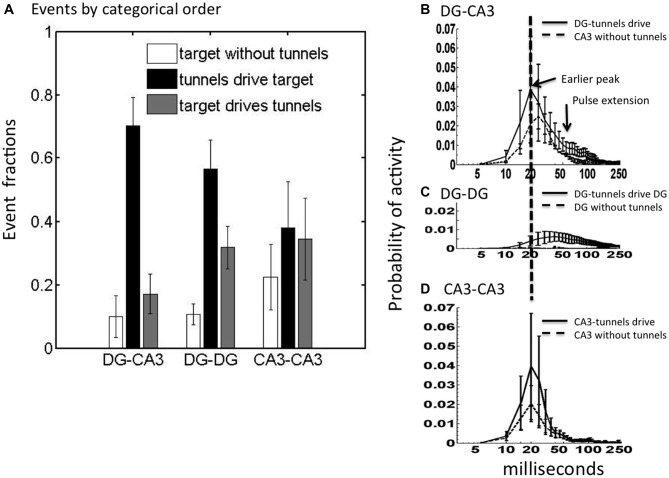
**CA3 is most frequently driven by tunnel activity with altered activity dynamics depending on inputs. (A)** In DG-CA3, the number of CA3 events driven by tunnels was higher than other categories (ANOVA *p* < 0.001) as was the case for DG-DG, but not CA3-CA3. Event fractions are the event counts in each category as a proportion of the total (*N* = 2782 for DG-CA3; 2280 for DG-DG; 1493 for CA3-CA3). **(B–D)** Post-event time histograms of spiking probability for CA3 (or target) events driven by tunnel and DG activity (solid) compared to target events without preceding tunnel activity (dashed; log scale). Note higher and earlier peak for CA3 when driven by tunnels than without tunnel inputs for native DG-CA3 networks. Target events were aligned at the start of each activity event. For each array, spike count was normalized to overall spike count in the target well. Normalized spike count (spiking probability) was averaged from five arrays. Error bars indicate ±SEM.

Spontaneous activity in the DG networks will include events transmitted axonally across the tunnels as well as many events reverberating in DG independent of tunnel transmission. Given that 84% of tunnel spikes propagate in the direction of DG to CA3, we postulated that most tunnel events were driven by DG and that many would drive activity in CA3. We segregated the relative fractions of CA3 events that occur in three categories: (a) CA3 without tunnel activity; (b) CA3 activity which follows and presumably is driven by tunnel activity (implying that DG is driving CA3); and (c) CA3 activity preceding and which presumably drives tunnel activity back toward DG. These statistics were averaged over five arrays for networks of DG apposed to CA3 (native relationship) as well as for control networks (DG-DG and CA3-CA3). Figure 3A shows that when CA3 was active without detected activity in the tunnels, which presumes little input from DG, the recorded activity across the CA3 compartment was low in number of events. When there was preceding tunnel activity, the CA3 compartment activity increased by a factor of seven. For CA3-CA3, events were almost equally frequent among these categories. In contrast, the largest category for both DG-CA3 and DG-DG was when activity in the target well was driven by activity in the tunnels, suggesting source-activated responses in the target. Of these tunnel-driven target events in DG-CA3 networks, we detected preceding DG source events about 45% of the time. The 70% of CA3 events driven by tunnel events (first black bar) was significantly higher in DG-CA3 networks than the 20% in which CA3 events drove tunnel events (ratio 3.5) or the 10% of CA3 events without tunnel input (Tukey-Kramer multiple comparison, *p* < 0.001). One-way ANOVAs within each of the three network configurations showed that the proportion of events differs in DG-CA3 (*p* < 0.001) and DG-DG (*p* < 0.001), but not in CA3-CA3, suggesting significant coupling for DG neurons but local CA3 activity largely unaffected by CA3 inputs through the tunnels. Together, these data suggest that in the DG-CA3 networks, the CA3 events are being preferentially driven by the activity propagating through the tunnels from DG.

To determine if the activity in CA3 was driven by inputs propagating through the tunnels from DG or if CA3 events were distinct from tunnel events, we constructed the mean envelope of responses as post-event time histograms, triggered on tunnel events. In DG-CA3 networks, if the tunnel activity drove CA3 activity, then we predicted that the spike dynamics of CA3 would be altered by that input. For DG-CA3 networks, if activity in the tunnels driven by DG influenced CA3, then the dynamics of the CA3 response would be different from that without tunnel input. Figure 3B shows both an increase in CA3 region peak amplitude and earlier time to peak at 20 ms for CA3 events driven by tunnel activity compared to a lower amplitude and delayed (25 ms) peak for CA3 activity without tunnel input from DG. Interestingly, tunnel input from DG also extended the envelope of the CA3 response from 50 to nearly 200 ms of the 250 ms window, suggesting more reverberation, compared to the CA3 envelope without tunnel input. For the control DG-DG, Figure 3C shows much lower network-wide activity, but of longer time to reach a peak around 40 ms and longer duration than DG-CA3 network activity. Isolated DG activity, without tunnel input from the other DG network was negligible and barely visible in the plot. Compared to DG-CA3 networks, CA3-CA3 networks in Figure 3D show similar probabilities of peak activity, but the CA3-CA3 networks were substantially lower in activity in the 50–100 ms time window compared to the temporal extension of activity for CA3 with inputs from DG at these times (Figure 3B).

### “First-to-Fire” Leader Neuron Sites in CA3

**Figure 4:**
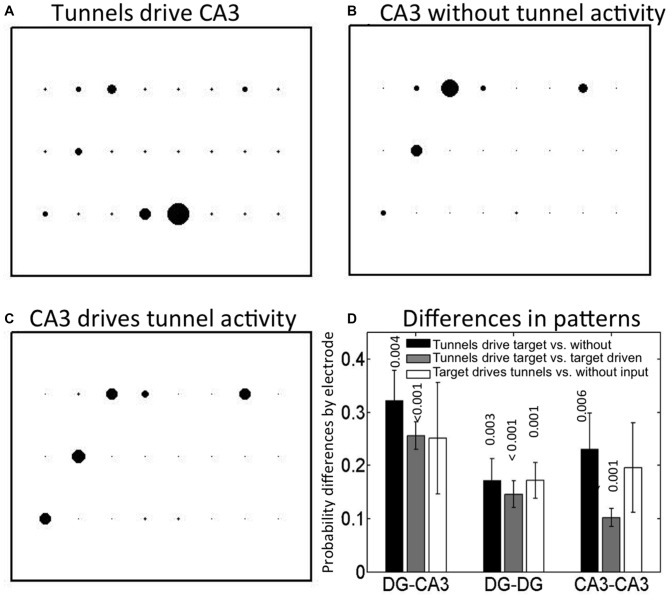
**Spatial map of first-to-fire CA3 probabilities (bubble diameters) depends on presence and timing of tunnel activity.** Different leader neuron probabilities among the 3 × 8 grid of CA3 electrodes in one DG-CA3 culture with **(A)** tunnels driving CA3 activity, **(B)** CA3 only, without tunnel activity, **(C)** CA3 driving tunnel activity. Tunnel side is below these depictions of activity in CA3. **(D)** Average spatial differences (distances) for five arrays by category. Differences: Black = tunnels driving target compared to target driving tunnels; gray = tunnels driving vs. target without tunnel activity; white = target drives tunnels vs. without input. Probabilities are indicated above each bar for the one-way *t*-test comparison to zero (no difference in pattern; *n* = 5).

Next, we asked whether the initiation site of activity was influenced by inputs through the tunnels from DG or was random. Figure 4A shows an example of the distribution of leader electrodes in one DG-CA3 network, where the diameter of the bubble reflects the relative probability that the electrode was first-to-fire. For this network, activity starts in row 3 (adjacent electrodes e53 or e43, i.e., column 5 or 4, row 3) in 82% of CA3 events driven by tunnels and DG events. In contrast, when CA3 events occurred without associated tunnel activity (Figure 4B), activity starts in either row 1 (e13) or row 2 (e22) 78% of the time. In Figure 4C, activity starts at multiple locations when tunnel events were driven by CA3 events (23% of the events in e31, 27% in e22, and 22% of the events in e13 or e17).

To determine how uniquely dependent the first-to-fire site was on tunnel activity, we computed the pairwise differences between categories (A-B, A-C or B-C) of the first-to-fire probability for the four most active sites in each category. We expected that a neuron in CA3 that always fired first, regardless of whether it was driven by a tunnel or not (A vs. B) would produce a small difference in these two high probabilities. Further, the difference would be insignificant when averaged over a number of arrays. Conversely, if the neuron in CA3 that fired first depended spatially on whether it was driven by a tunnel or not (A vs. B), the difference in probability at that electrode would be large. Further, the differences might be significant when averaged over a number of arrays. Thus, Figure 4D depicts these categorical differences in first-to-fire probabilities and indicates whether the means are significantly different from zero (same electrode, but different tunnel activity category) for five arrays. The spatial distribution of leader electrodes in CA3 (or target) events when driven by tunnel events was different from those without associated tunnel events (black bar, *p* = 0.004) or those in which CA3 events drive tunnel activity (gray bar, *p* < 0.001). The third category of events that initiated in CA3, driving tunnels vs. no tunnel activity (white bar) was more variable and did not reach the adjusted significance criterion for *p* (three comparisons) < 0.017. For DG-DG, all three categories were separable by spatial pattern, indicating that DG events initiate at different but distinct locations within each culture depending on whether the region is driven by tunnel activity, drives tunnel activity, or is temporally isolated from the communicating tunnels, but distance measures were about 40% lower than for DG-CA3 categories. For CA3-CA3, inputs also altered first-to-fire activation location. Although the patterns of activity in the target for DG-DG and CA3-CA3 controls were separable, the separation distance trended lower than those of DG-CA3. Together, these results indicate that spatial patterns of activity in CA3 (or target network) are controlled by the tunnel inputs from DG (or source).

### Dependence of CA3 Activity on Number of Tunnel Inputs

**Figure 5:**
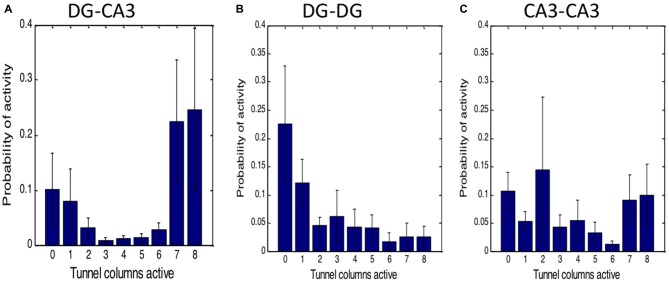
**High number of tunnels active simultaneously for DG driving CA3 activity in contrast to homologous controls. (A)** Robust connection of DG through tunnels to CA3. Zero tunnels active represents CA3 or target activity without tunnel input. In homologous networks of **(B)** DG-DG or **(C)** CA3-CA3, activity is largely independent of tunnel activity. Probabilities as a fraction of total events in target well (*n* = 5 networks each).

We also assessed the fraction of the tunnel events driving CA3 events to determine whether the amount of activation from the tunnels was indicative of the type of events occurring in CA3. The first bar of Figure 5A indicates about 10% of the CA3 events occur without detected tunnel input. The remaining bars were heavily skewed toward 7 or 8 (full) tunnels actively driving CA3 activity. When tunnel inputs from the DG network were counted, 41% of the 6, 7 or 8 tunnel-CA3 events were driven by DG events (data not shown). This 41% value also indicates that the tunnels are not just sampling activity from a DG network that is active all the time, but that specific kinds of DG activity such as spatial or temporal patterns activate tunnel axons and in turn activate CA3. In contrast, Figure 5B shows that no detected tunnel input to DG from the apposing DG network occurred 22% of the time (first bar) and a distribution weighted toward few tunnel inputs, the opposite of DG-CA3 networks. Of all the DG events driven by tunnel activity, the opposing DG network drove these tunnels 39% of the time. In CA3-CA3 networks, tunnel count was more evenly distributed (Figure 5C) and 54% of the target well events were driven by source events. Overall, the largest amplitude category of CA3 activation suggests self-wiring of a nearly simultaneous all-or-nothing group of DG inputs through the tunnels.

### Logistic Regression by Number of Tunnel Inputs Predicts CA3 Activity

**Figure 6:**
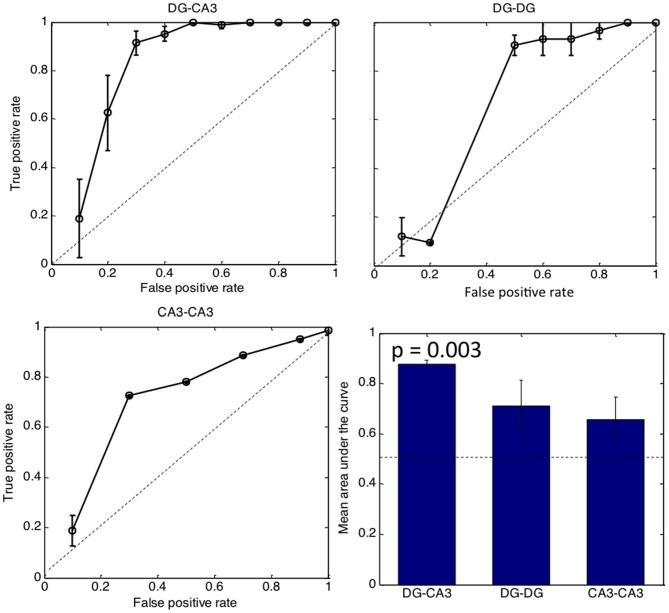
**Tunnel count predicts CA3 activity by logistic regression but not in control networks.** The bar graph indicates area under the curve for each category with the dashed line representing no predictive ability. Only the DG-CA3 bar is significantly better than chance. (*n* = 3 most active arrays in each configuration).

Figure 6 shows the effectiveness of the number of tunnels to predict CA3 activity by logistic regression. The larger the area under the curve, the better the reliability of tunnel number in coding for CA3 activity. The diagonal represents no predictive power. Good predictive power occurs with a high true positive (*y*-axis) representing sensitivity with a low false positive. False positive is related to the complementary term specificity, calculated as 1-false positive rate. Logistic regression indicated that CA3 activity is predicted by the number of tunnel inputs from DG with 90% sensitivity (*ad hoc* threshold) and 70% specificity (1-false positive rate). Other classifiers of maximum spike rate, tunnel activation order and presence or absence of tunnel activity were nearly as good as the tunnel count curve shown for DG-CA3 (not shown). In contrast, the 90% sensitivity level was reached at only 50% specificity in DG-DG networks and only 30% specificity in CA3-CA3 networks. The bar graph indicates a significant area-under the curve for the three most active DG-CA3 arrays (*p* = 0.003; one-tailed *t*-test for fraction greater than 0.5) while DG-DG and CA3-CA3 networks were not significantly different from 0.5. Together, Figures 5, 6, indicate a predictive relationship between tunnel count and target activity in DG-CA3, i.e., that propagation of activity through the tunnels is highly dependent on the native and appropriate (i.e., DG-CA3) source-target relationship.

### Distinct CA3 Spatial Patterns that Repeat Three or More Times

**Figure 7:**
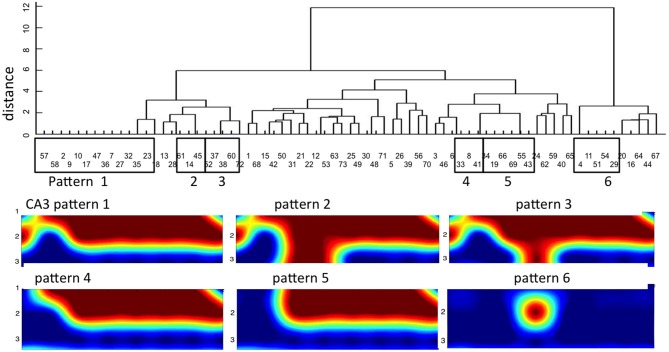
**Dendrogram clustering reveals distinct temporal-spatial distributions of CA3 activity for 8 tunnel inputs from DG.** X-axis of event order indicates mostly non-sequential repeat patterns. Y-axis measures distance with zero equal to identical spatial patterns in CA3. Of 73 timed patterns, six groups repeat three or more times (boxed). The spatial distributions of these patterns are shown below on the 3 × 8 grid of CA3 electrodes.

To establish that activity in CA3 was not random or unpredictable, we searched for motifs as repetitive temporal or spatial events. We begin identification of spatial motifs by coding each CA3 electrode as active or inactive in each event propagating through the tunnels from DG (binary coding). Next we sorted the variety of spontaneous activity patterns across the 22 target well electrodes that were evoked by tunnel activity. Random independence would predict 2^22^ (4 × 10^6^) possible combinations of spatial patterns. Instead, in one 3 min recording from a single DG-CA3 culture, dendrogram clustering showed only 42 patterns of which six motifs repeated three or more times (Figure 7). Figure 7 also shows the nature of these spatial motifs in CA3 with activation of a large fraction of all the sites monitored in five of the six patterns that repeated three or more times. The first three patterns differ only in row 3, with 0, 2 or 1 electrode detecting activity, respectively. The unusual response six was associated with inhibition of a large number of recording sites, suggesting that this electrode reported the activity of an inhibitory neuron that shut down the rest of the network in the well.

### Spatial-Temporal Tunnel Coding of Activity Motifs in CA3

**Figure 8:**
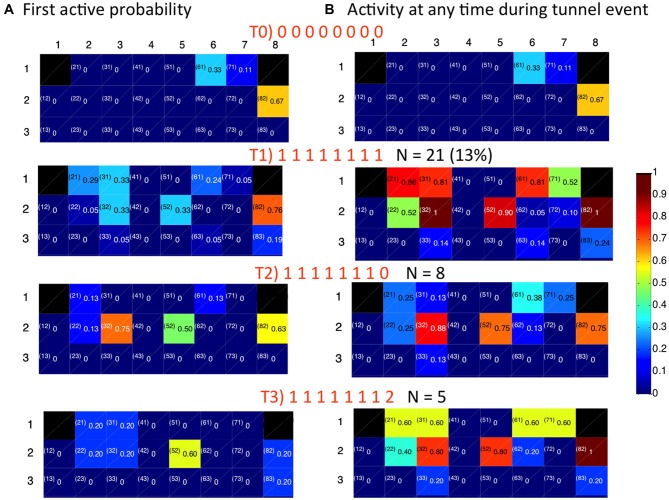
**Spatial distribution of activity for indicated temporal patterns of activity in 8 tunnels depend on timing and pattern of tunnel events (0 = tunnel inactive, 1 = first event (often simultaneous), 2 = second delayed event). (A)** First to fire activation patterns in target CA3. Numbers are the fraction of events that an electrode was the first to be active. **(B)** Extent of activation in CA3. Numbers are the fraction of time each electrode is active during the indicated pattern of tunnel activation.

Figure 8 shows the association of CA3 repeating motifs with four distinct patterns of tunnel activation from DG (T0, T1, T2, T3) from another DG-CA3 network. T0 is the case where no tunnels are active, while the others differ only in activity in tunnel 8: T1 has a tunnel eight event contemporaneous with events in the other tunnels; in T2 there is no activity in tunnel 8; and in T3, the tunnel 8 event is delayed. Figure 8A shows the probabilities that an indicated electrode is the first-to-fire in CA3. Figure 8B shows the individual electrodes that are active at any time during inputs from DG via the tunnels. The first row shows probabilities of CA3 activity when there are no inputs from the tunnels (T0); only three electrodes in CA3 are active in this case. In T1, all the tunnels are simultaneously active, associated with more activity in CA3 rows 1 and 2 and some activity in row 3. This pattern repeated 21 times in our 3 min recording, accounting for 13% of the observed patterns. Five percent of the patterns were associated with 7 of the 8 tunnel electrodes being active (T2) and lower probabilities of row 1 activation in CA3. The next most frequent pattern (T3 at 3%) was more exclusive in first activating e52 and not e82 coded by a 32 ms delay in tunnel eight activity (fourth row, Figure 8A). With this T3 pattern of stimulus, CA3 row 1 was again activated (Figure 8B). Together, these results indicate discrete input coding from DG into predictable patterns of CA3 activation.

**Figure 9:**
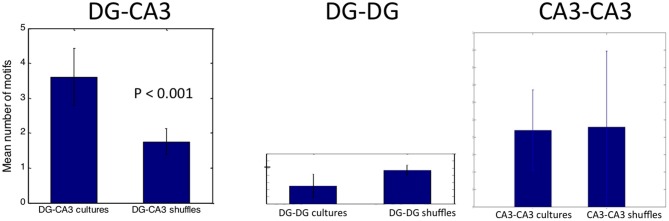
**Shuffle test for significant spatial-temporal pattern of activity in target driven by 7, 8 tunnels.** Note that only the DG-CA3 network has significantly more motifs than chance shuffling.

To establish the statistical significance of these motifs, we averaged the motif count from each of five DG-CA3 networks, compared to motif counts if the electrode locations were shuffled. Figure 9 shows an average 3.6 motifs repeating three or more times detected in 3 min (1.2/min), more than twice the frequency obtained in shuffled data (*p* < 0.001). Comparisons to control DG-DG and CA3-CA3 networks revealed a lower number of motifs that were not significant compared to shuffled data.

In Table 1, we compare our motif results to other publications. Our rate of detection is within the range reported by others for motifs that repeat three or more times *in vivo* and *in vitro* studies, but this rate is highly dependent on temporal resolution. Most significantly, our studies report the involvement of 14–17 recorded sites in five of the six patterns in our motifs (Figure 7), much larger than the mean 2–7 units reported by others.

**Table 1:** **Motifs in the brain and cultured neuron networks**.

Subject	Resolution (ms)	Repeats	Motifs/min	Shuffled motifs/min	Number of neurons (units) in motif (range)	Motif length (ms)	Method	Reference
Slice mouse primary visual cortex EPSC’s from 2 neurons	2	≥3	7		2		Covariance 20 ms	Ikegaya et al. (2004)
-same-	1	≥3	0.6		2		Covariance 20 ms	
Ca++ transients, mouse primary visual/prefrontal cortex	1000		1.8	50%			Covariance 1 s	
Mouse frontal cortex culture	10		18	1.2	3 (3–6)		Joint entropy	Bettencourt et al. (2007)
Rat cortical culture 21 div	1	≥2	0.8	0.3	2 (2–13)	125	Template match	Rolston et al. (2007)
-same-	1	≥3	0.1		3 (3–6)	105	-same-	
Mouse cortical culture13–15 div	2	≥2	0.3	n/a	5	100	Template match	Sun et al. (2010)
		≥3	0.08		3 (2–4)	100
Rat Entorhinal-hippocampal slice culture, Calcium imaging	2	≥2	40	5			Pairwise corrrelogram	Matsumoto et al. (2013)
	4	≥2	300	50	6 (3–15)	60 (40–280)	-same-	
Freely moving mouse CA1	6		40%	13%	7 (4–9)	143	Template or pairwise rank	Stark et al. (2015)
Dissociated rat DG connected to CA3	32	≥3	1.2	0.6	14–17		Template	This work

*n/a = not available but significantly less than unshuffled data*.

## Discussion

The tunnel design of our engineered reconstruction of two hippocampal subregions, namely, DG and CA3, enabled us to monitor the self-wired connections between the sub-networks. Previously, we showed that our 2-compartment DG-CA3 networks produced separable spike and burst dynamics, spike direction information and biomarkers to validate that the subregions maintained their identities (Brewer et al., 2013). Here the result is extended to dependence of network activity on connectivity. Highly polarized spontaneous propagation of over 80% of the activity that proceeded in the native direction from DG to CA3 indicates self-wiring capabilities intrinsic to these neurons. Differences from control networks of DG-DG and CA3-CA3 emerged when the relationships of target CA3 activity were categorized with regard to inputs from the apposing network through the tunnels. As compared to no apparent driving input from DG, CA3 activity driven by DG was greater in amplitude and extended in time, similar to the *in vivo* burst time to peak in CA3 (20–40 ms) and pulse extension with five input spikes (Miles and Wong, 1986). Also, when DG drove the activity in CA3, the pattern of first-to-fire CA3 neurons was different from when CA3 was without input, which, in turn, was separable from when CA3 appeared to drive DG back through the tunnels. A clustering algorithm was used to reveal repeating spatial patterns of activity in the CA3 network that were associated with temporal patterns of activation through the tunnels from DG. Together, these results add to evidence for coding of patterns of information as distributed activity in the CA3, even when driven by spontaneous activation. These significantly repeated motifs in CA3 are shown for the first time to be activated by specifically timed, often simultaneous inputs from our reconstructed DG network.

### Leader Neurons

Leader neurons have been noted previously in dissociated hippocampal and cortical cultures (Eytan and Marom, 2006; Eckmann et al., 2008; Stegenga et al., 2008), but the pattern of stimulation for the leader neuron to spontaneously initiate a burst of network-wide activity has not been identified. Eckmann et al. (2008) nicely quantified leader persistence to fall off logarithmically with time (half-life of about 24 h). They also identified a smaller preburst network activated within the vicinity of the leader neuron, but the nature of these inputs to trigger the leader was not identified. Stimulation of their rat hippocampal networks generally lowered the probability of prior spontaneous leadership. Stegenga et al. (2008) observed stable, network-wide spatio-temporal burst envelopes associated with first-to-fire sites. Here, we identified specific kinds of spontaneous inputs that alter which neurons fire first. Specifically, we found different leader neurons in CA3 events when a maximum number of inputs came through the tunnels from the DG network, compared to CA3 events without tunnel-DG inputs or back-propagation from CA3 to the tunnels.

### Motifs

Table 1 summarizes the work of several labs to identify and characterize the *in vivo* and *in vitro* motifs. Sporns and Kötter (2004) were the first to define brain motifs in macaque and cat cortex as discrete spatial patterns of connectivity significantly different from random connections of all the nodes. They further distinguished physically connected structural motifs from electrically functional motifs. However, Ikegaya et al. (2004) was the first to report timed repeating functional motifs that they called synfire chains. Rolston et al. (2007) was the first to show from dissociated cortical neurons in culture that precisely timed repeated patterns are an emergent feature of self-organizing networks and Sun et al. (2010) more precisely characterized these motifs both temporally and spatially. More recently, Matsumoto et al. (2013) studied calcium signals from cultured rat hippocampal slices to find replay of repeating patterns during sharp-wave ripples in the local field potentials. Stark et al. (2015) extended these studies into freely moving mouse CA1 to identify interneurons as the source of the ripple that temporally organized spike sequences within theta wave oscillations. Since detection of repeated activity depends on time resolution, recording time and overall activity, we extracted the motifs per min at a given time resolution for each report. Importantly, these repeating patterns were either two or three times what could be expected by chance shuffling for that level of activity and length of motif. Our results of 1.2 motifs/min for ≥3 repeats and double the chance probability is in line with Ikegaya et al. (2004) for calcium transients, but less motifs than Ikegaya et al. (2004) for EPSC’s in mouse slice, Bettencourt et al. (2007) for joint entropy and Matsumoto et al. (2013) by pairwise correlation. Notably, our rate of observation of spatial-temporal motifs for three repeats exceeds those of Rolston et al. (2007) and Sun et al. (2010), possibly because we focused on the highly recurrent connections in CA3 that were specifically activated by DG through the tunnels. Our results of 14–17 units in each motif (Figure 7) far exceeds those reported by others, possibly for the same reasons.

### Limitations of Our Reconstructed Networks

Neurons removed from their native brain inputs and anatomical subregions might homogenize in a common culture medium. Our findings here of highly polarized transmission in the native DG-CA3 direction and differences in burst dynamics suggest intrinsic self-wiring capacity. Our previous studies of these same networks indicated maintenance of enrichment of gene expression for hippocampal subregions as well as subregional differences in inhibitory neuron and astroglial composition (Brewer et al., 2013). Obviously, we have destroyed the uniquely lamellar organization of the hippocampal subregions and removed them from their native inputs. Our native 3:1 ratio of DG to CA3 neurons is certain to affect channel spike rates compared to a 1:1 or other ratios, but the log-log distribution of ISI were not different in slope between DG and CA3. It seems less likely that the ratio would affect other emergent properties such as tunnel activity patterns with inputs from DG that can specify CA3 firing patterns. Nevertheless, our studies suggest a self-wiring capacity intrinsic to the DG and CA3 subregions that can produce inter-regional connectivity, first-to-fire properties and even some measures of repeated activity patterns. Roxin et al. (2008) elegantly demonstrate that spatial-temporal motifs can be modeled by Poisson statistics to approximate the motifs reported by Ikegaya et al. (2004). We agree that motifs emerge by chance from large data sets and high rates of activity, but purport that significant motif counts above those found by shuffling the data and their dependence on input patterns decrease the likelihood of a chance finding.

## Conclusion

This reconstruction of two hippocampal subregions with spontaneous inter-regional communication demonstrates the utility of robust monitoring through our engineered tunnels aligned to electrodes. The high signal to noise ratio in these tunnels enables detection of spikes from multiple axons (Pan et al., 2014). This arrangement enabled detection of spontaneous input-dependent repeatable coding of distributed activity in CA3 networks driven by the engineered inputs from DG networks that we failed to detect without knowledge of which source events were the driving stimuli for the target. The spontaneous functional configurations at measured times of activation (motifs) emerged from anatomically accurate feed-forward connections. Future work will reveal additional insights about repeating spatial-temporal motifs from stimulation and similar analysis of other hippocampal subregion pairs, CA3-CA1, CA1-EC and EC-DG. Our approach should be useful for monitoring development and altered plasticity in cultured networks.

## Author Contributions

AB, BCW, TBDeM, GJB and BCW designed the experiments. AB and TBDeM analyzed the original recordings. HD analyzed spike and burst dynamics. All authors contributed to interpretation. AB and GJB wrote the article. GJB, BCW and TBD edited the manuscript.

## Conflict of Interest Statement

The authors declare that the research was conducted in the absence of any commercial or financial relationships that could be construed as a potential conflict of interest.

## Footnotes

^1^http://tuvalu.santafe.edu/~aaronc/powerlaws/

## References

[B1] Bettencourt L. M. A. Stephens G. J. Ham M. I. Gross G. W. (2007). Functional structure of cortical neuronal networks grown *in vitro*. Phys. Rev. E Stat. Nonlin. Soft Matter Phys. 75:021915. 10.1103/physreve.75.021915 17358375

[B2] Bisio M. Bosca A. Pasquale V. Bedondini L. Chiappalone M. (2014). Emergence of bursting activity in connected neuronal sub-populations. PLoS One 9:e107400. 10.1371/journal.pone.0107400 25250616 PMC4175468

[B3] Bologna L. L. Pasquale V. Garofalo M. Gandolfo M. Baljon P. L. Maccione A.. (2010). Investigating neuronal activity by SPYCODE multi-channel data analyzer. Neural Netw. 23, 685–697. 10.1016/j.neunet.2010.05.002 20554151

[B4] Braitenberg V. (1981). “Anatomical basis for divergence and intergration in the cerebral cortex,” in Advances in Physiology Education (Vol. 16), eds Grastyban E. Molnbar P. (Elmsford, NY: Pergamon Press), 411–419.

[B5] Brewer G. J. Boehler M. D. Jones T. T. Wheeler B. C. (2008). NbActiv4 medium improvement to Neurobasal/B27 increases neuron synapse densities and network spike rates on multielectrode arrays. J. Neurosci. Methods 170, 181–187. 10.1016/j.jneumeth.2008.01.009 18308400 PMC2393548

[B6] Brewer G. J. Boehler M. D. Leondopulos S. Pan L. Alagapan S. Demarse T. B.. (2013). Toward a self-wired active reconstruction of the hippocampal trisynaptic loop: DG-CA3. Front. Neural Circuits 7:165. 10.3389/fncir.2013.00165 24155693 PMC3800815

[B7] Buzsáki G. Mizuseki K. (2014). The log-dynamic brain: how skewed distributions affect network operations. Nat. Rev. Neurosci. 15, 264–278. 10.1038/nrn3687 24569488 PMC4051294

[B8] Clauset A. Shalizi C. R. Newman M. E. J. (2009). Power-law distributions in empirical data. SIAM Rev. 51, 661–703. 10.1137/070710111

[B9] Claverol-Tinturé E. Cabestany J. Rosell X. (2007). Multisite recording of extracellular potentials produced by microchannel-confined neurons *in vitro*. IEEE Trans. Biomed. Eng. 54, 331–335. 10.1109/tbme.2006.880903 17278590

[B11] Eckmann J.-P. Jacobi S. Marom S. Moses E. Zbinden C. (2008). Leader neurons in population bursts of 2D living neural networks. New J. Physics 10:015011 10.1088/1367-2630/10/1/015011

[B12] Eytan D. Marom S. (2006). Dynamics and effective topology underlying synchronization in networks of cortical neurons. J. Neurosci. 26, 8465–8476. 10.1523/jneurosci.1627-06.2006 16914671 PMC6674346

[B13] Ham M. I. Bettencourt L. M. McDaniel F. D. Gross G. W. (2008). Spontaneous coordinated activity in cultured networks: analysis of multiple ignition sites, primary circuits and burst phase delay distributions. J. Comput. Neurosci. 24, 346–357. 10.1007/s10827-007-0059-1 18066657

[B14] Ikegaya Y. Aaron G. Cossart R. Aronov D. Lampl I. Ferster D.. (2004). Synfire chains and cortical songs: temporal modules of cortical activity. Science 304, 559–564. 10.1126/science.1093173 15105494

[B15] Kanagasabapathi T. T. Franco M. Barone R. A. Martinoia S. Wadman W. J. Decré M. M. (2013). Selective pharmacological manipulation of cortical-thalamic co-cultures in a dual-compartment device. J. Neurosci. Methods 214, 1–8. 10.1016/j.jneumeth.2012.12.019 23305774

[B16] Maccione A. Gandolfo M. Massobrio P. Novellino A. Martinoia S. Chiappalone M. (2009). A novel algorithm for precise identification of spikes in extracellularly recorded neuronal signals. J. Neurosci. Methods 177, 241–249. 10.1016/j.jneumeth.2008.09.026 18957306

[B17] Maeda E. Kuroda Y. Robinson H. P. Kawana A. (1998). Modification of parallel activity elicited by propagating bursts in developing networks of rat cortical neurones. Eur. J. Neurosci. 10, 488–496. 10.1046/j.1460-9568.1998.00062.x 9749711

[B18] Maeda E. Robinson H. P. Kawana A. (1995). The mechanisms of generation and propagation of synchronized bursting in developing networks of cortical neurons. J. Neurosci. 15, 6834–6845. 7472441 10.1523/JNEUROSCI.15-10-06834.1995 PMC6578010

[B19] Matsumoto K. Ishikawa T. Matsuki N. Ikegaya Y. (2013). Multineuronal spike sequences repeat with millisecond precision. Front. Neural Circuits 7:112. 10.3389/fncir.2013.00112 23801942 PMC3689151

[B20] Miles R. Wong R. K. (1986). Excitatory synaptic interactions between CA3 neurones in the guinea-pig hippocampus. J. Physiol. 373, 397–418. 10.1113/jphysiol.1986.sp016055 3018233 PMC1182545

[B21] Newman M. E. J. (2005). Power laws, Pareto distributions and Zipf’s law. Contemp. Phys. 46, 323–351. 10.1080/00107510500052444

[B22] Pan L. Alagapan S. Franca E. Brewer G. J. Wheeler B. C. (2011). Propagation of action potential activity in a predefined microtunnel neural network. J. Neural Eng. 8:046031. 10.1088/1741-2560/8/4/046031 21750372 PMC3213028

[B23] Pan L. Alagapan S. Franca E. DeMarse T. Brewer G. J. Wheeler B. C. (2014). Large extracellular spikes recordable from axons in microtunnels. IEEE Trans. Neural Syst. Rehabil. Eng. 22, 453–459. 10.1109/TNSRE.2013.2289911 24240004 PMC4013201

[B24] Pan L. Alagapan S. Franca E. Leondopulos S. S. DeMarse T. B. Brewer G. J.. (2015). An *in vitro* method to manipulate the direction and functional strength between neural populations. Front. Neural Circuits 9:32. 10.3389/fncir.2015.00032 26236198 PMC4500931

[B25] Patolsky F. Timko B. P. Yu G. Fang Y. Greytak A. B. Zheng G.. (2006). Detection, stimulation and inhibition of neuronal signals with high-density nanowire transistor arrays. Science 313, 1100–1104. 10.1126/science.1128640 16931757

[B26] Poli D. Pastore V. P. Massobrio P. (2015). Functional connectivity in *in vitro* neuronal assemblies. Front. Neural Circuits 9:57. 10.3389/fncir.2015.00057 26500505 PMC4595785

[B27] Rolston J. D. Wagenaar D. A. Potter S. M. (2007). Precisely timed spatiotemporal patterns of neural activity in dissociated cortical cultures. Neuroscience 148, 294–303. 10.1016/j.neuroscience.2007.05.025 17614210 PMC2096414

[B28] Roxin A. Hakim V. Brunel N. (2008). The statistics of repeating patterns of cortical activity can be reproduced by a model network of stochastic binary neurons. J. Neurosci. 28, 10734–10745. 10.1523/JNEUROSCI.1016-08.2008 18923048 PMC6671336

[B29] Sandler R. A. Song D. Hampson R. E. Deadwyler S. A. Berger T. W. Marmarelis V. Z. (2015). Hippocampal closed-loop modeling and implications for seizure stimulation design. J. Neural Eng. 12:056017. 10.1088/1741-2560/12/5/056017 26355815 PMC5030843

[B30] Segev R. Baruchi I. Hulata E. Ben-Jacob E. (2004). Hidden neuronal correlations in cultured networks. Phys. Rev. Lett. 92:118102. 10.1103/physrevlett.92.118102 15089177

[B31] Sporns O. Kötter R. (2004). Motifs in brain networks. PLoS Biol. 2:e369. 10.1371/journal.pbio.0020369 15510229 PMC524253

[B32] Stark E. Roux L. Eichler R. Buzsáki G. (2015). Local generation of multineuronal spike sequences in the hippocampal CA1 region. Proc. Natl. Acad. Sci. U S A 112, 10521–10526. 10.1073/pnas.1508785112 26240336 PMC4547251

[B33] Stegenga J. Le Feber J. Marani E. Rutten W. C. (2008). Analysis of cultured neuronal networks using intraburst firing characteristics. IEEE Trans. Biomed. Eng. 55, 1382–1390. 10.1109/TBME.2007.913987 18390329

[B34] Sun J. J. Kilb W. Luhmann H. J. (2010). Self-organization of repetitive spike patterns in developing neuronal networks *in vitro*. Eur. J. Neurosci. 32, 1289–1299. 10.1111/j.1460-9568.2010.07383.x 20846326

[B35] Taylor A. M. Jeon N. L. (2011). Microfluidic and compartmentalized platforms for neurobiological research. Crit. Rev. Biomed. Eng. 39, 185–200. 10.1615/CritRevBiomedEng.v39.i3.20 21967302

[B36] Taylor A. M. Rhee S. W. Tu C. H. Cribbs D. H. Cotman C. W. Jeon N. L. (2003). Microfluidic multicompartment device for neuroscience research. Langmuir 19, 1551–1556. 10.1021/la026417v 20725530 PMC2923462

[B37] van Pelt J. Wolters P. S. Corner M. A. Rutten W. L. Ramakers G. J. (2004). Long-term characterization of firing dynamics of spontaneous bursts in cultured neural networks. IEEE Trans. Biomed. Eng. 51, 2051–2062. 10.1109/tbme.2004.827936 15536907

[B38] Volman V. Baruchi I. Ben-Jacob E. (2005). Manifestation of function-follow-form in cultured neuronal networks. Phys. Biol. 2, 98–110. 10.1088/1478-3975/2/2/003 16204862

[B39] Wagenaar D. A. Pine J. Potter S. M. (2006). An extremely rich repertoire of bursting patterns during the development of cortical cultures. BMC Neurosci. 7:11. 10.1186/1471-2202-7-11 16464257 PMC1420316

[B40] Wang L. Riss M. Buitrago J. O. Claverol-Tinturé E. (2012). Biophysics of microchannel-enabled neuron-electrode interfaces. J. Neural Eng. 9:026010. 10.1088/1741-2560/9/2/026010 22333069

