# Supplementary figures and images for: Repeating Spatial-Temporal Motifs of CA3 Activity Dependent on Engineered Inputs from Dentate Gyrus Neurons in Live Hippocampal Networks
Source: Front Neural Circuits. 2016 Jun 28;10:45. doi: 10.3389/fncir.2016.00045 (PMC4923256; doi:10.3389/fncir.2016.00045)

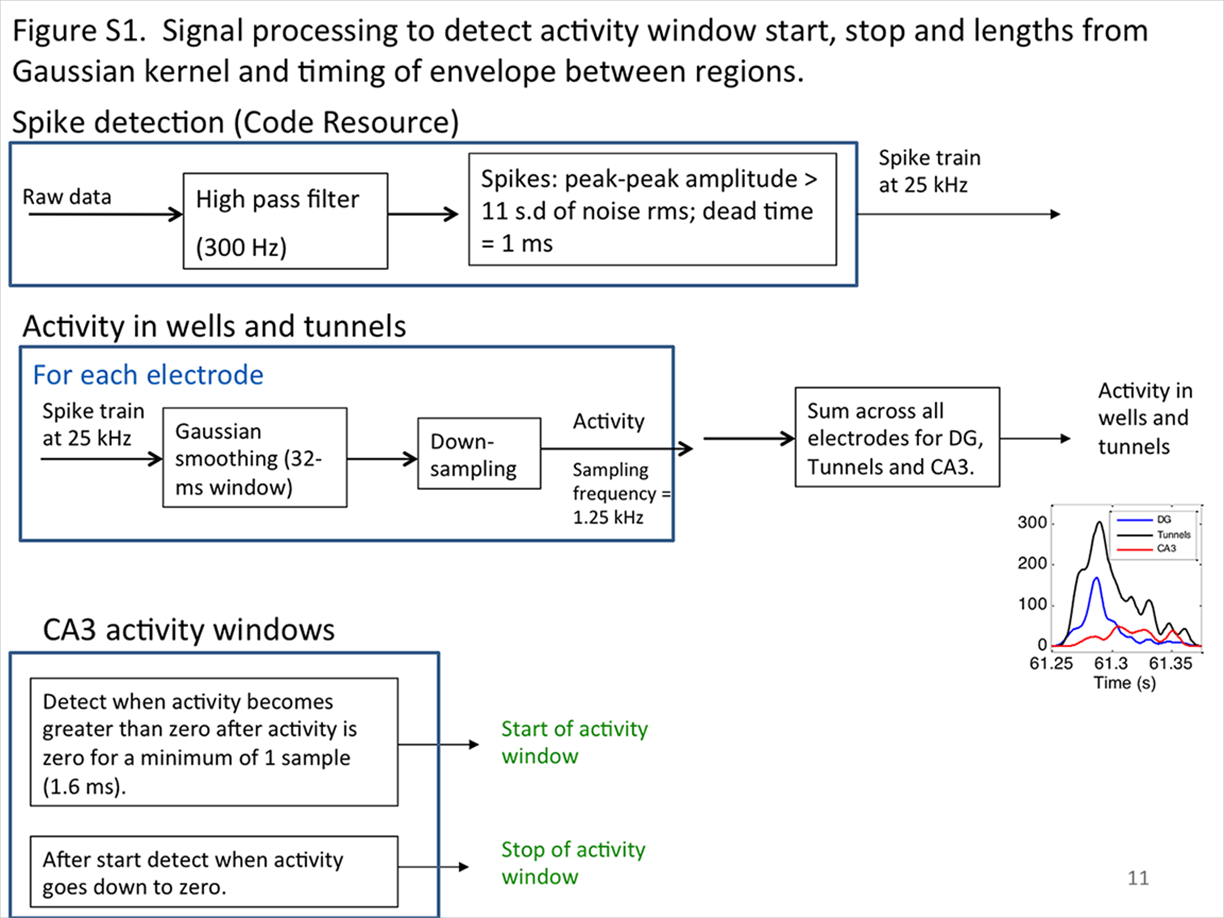

Supplement: Supplementary file 1 [file Image_1.tif]
